# *N*-(Trimethyl­sil­yl)methane­sulfonamide

**DOI:** 10.1107/S1600536810050336

**Published:** 2010-12-11

**Authors:** Andrew R. McWilliams, Sossina Gezahegna, Alan J. Lough

**Affiliations:** a Department of Chemistry & Biology, Ryerson University, Toronto, Ontario, Canada M5B 2K3; b Department of Chemistry, University of Toronto, Toronto, Ontario, Canada M5S 3H6

## Abstract

There are two mol­ecules in the asymmetric unit of the title compound, C_4_H_13_NO_2_SSi. In the crystal, mol­ecules are linked *via* inter­molecular N—H⋯O hydrogen bonds, forming chains along [001]. The crystal studied was an inversion twin, the refined ratio of twin domains being 0.61 (9):0.39 (9).

## Related literature

For the original synthesis of the title compound, see: Roy (1993 ▶). For the synthetic application of the title compound, see: Roy *et al.* (1993 ▶). For related structures, see: Ni *et al.* (1995 ▶); Chunechom *et al.* (1998 ▶).
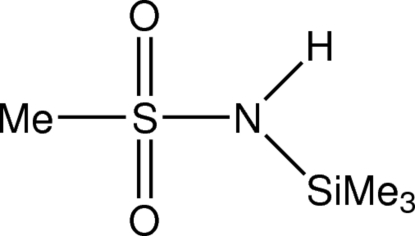


## Experimental

### 

#### Crystal data

C_4_H_13_NO_2_SSi
*M* *_r_* = 167.30
Monoclinic, 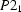

*a* = 8.2827 (4) Å
*b* = 10.9513 (5) Å
*c* = 9.6201 (3) Å
β = 92.536 (2)°
*V* = 871.75 (6) Å^3^
*Z* = 4
Mo *K*α radiation
μ = 0.45 mm^−1^
*T* = 150 K
0.32 × 0.25 × 0.24 mm

#### Data collection

Nonius KappaCCD diffractometer
Absorption correction: multi-scan (*SORTAV*; Blessing 1995 ▶) *T*_min_ = 0.830, *T*_max_ = 0.931
6920 measured reflections
4894 independent reflections
4195 reflections with *I* > 2σ(*I*)
*R*_int_ = 0.030

#### Refinement

*R*[*F* ^2^ > 2σ(*F* ^2^)] = 0.039
*wR*(*F* ^2^) = 0.094
*S* = 1.05
4894 reflections
170 parameters
2 restraints
H atoms treated by a mixture of independent and constrained refinement
Δρ_max_ = 0.41 e Å^−3^
Δρ_min_ = −0.49 e Å^−3^
Absolute structure: Flack (1983 ▶), 1787 Friedel pairs
Flack parameter: 0.39 (9)

### 

Data collection: *COLLECT* (Nonius, 2002 ▶); cell refinement: *DENZO-SMN* (Otwinowski & Minor, 1997 ▶); data reduction: *DENZO-SMN*; program(s) used to solve structure: *SIR92* (Altomare *et al.*, 1994 ▶); program(s) used to refine structure: *SHELXTL* (Sheldrick, 2008 ▶); molecular graphics: *PLATON* (Spek, 2009 ▶); software used to prepare material for publication: *SHELXTL*.

## Supplementary Material

Crystal structure: contains datablocks global, I. DOI: 10.1107/S1600536810050336/si2314sup1.cif

Structure factors: contains datablocks I. DOI: 10.1107/S1600536810050336/si2314Isup2.hkl

Additional supplementary materials:  crystallographic information; 3D view; checkCIF report

## Figures and Tables

**Table 1:** Hydrogen-bond geometry (Å, °)

*D*—H⋯*A*	*D*—H	H⋯*A*	*D*⋯*A*	*D*—H⋯*A*
N1*B*—H1*NB*⋯O2*A*	0.81 (2)	2.11 (2)	2.917 (3)	173 (3)
N1*A*—H1*NA*⋯O2*B*^i^	0.81 (2)	2.12 (2)	2.925 (3)	177 (3)

Symmetry code: (i) 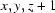.

**Fig. 1.**
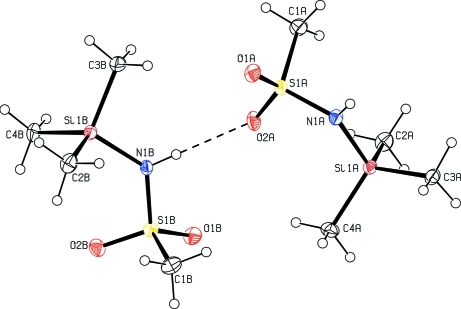
The asymmetric unit of title compound showing 30% probability ellipsoids. The dashed line indicates a hydrogen bond.

**Fig. 2.**
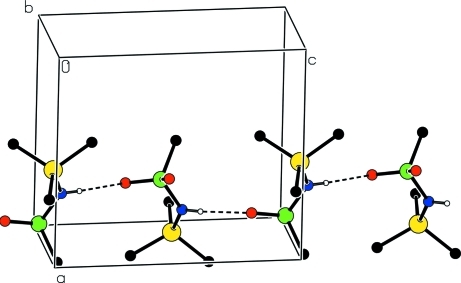
Part of the crystal structure showing hydrogen bonds as dashed lines.

## supplementary crystallographic information

### Comment

*N*-trimethylsilylmethylsulfonamide, a key intermediate in the synthesis of polyoxothiazenes (Roy *et al.*, 1993) and polythionylphosphazenes (Chunechom *et al.*, 1998), was prepared *via* the reaction of methanesulfonyl chloride and hexamethyldisilazane (Roy, 1993). The asymmetric unit of the title compound, which contains two independent molecules, is shown in Fig. 1. The S—N bond distances in each molecule are intermediate between a typical S—N single bond (1.74 Å) and a typical S=N double bond (1.54 Å), (Ni *et al.*, 1995) suggesting the presence of some π-bonding between the sulfur and nitrogen atoms. The S—N—Si bond angles of 127.83 (14)° and 128.59 (14)Å are larger than might be expected, in terms of hybridization priciples, for either a tetrahedral or trigonal planar geometry about the nitrogen atom. In the crystal structure, molecules are linked *via* intermolecular N—H···O hydrogen bonds to form one-dimensional chains along [001] (Fig. 2).

### Experimental

The title compound was prepared *via* addition of methanesulfonyl chloride (7 ml, 102.5 mmol) to a three-necked round-bottom flask equipped with a magnetic stirring bar, gas inlet, reflux condenser and a rubber septa under an inert N_2_ atmosphere. Hexamethyldisilazane (20 ml, 103.1 mmol) was added drop wise over 10 minutes with stirring at ambient temperatures. The flask was then placed into an oil bath and the reaction mixture heated to 363–373 K to initiate the reaction. The temperature of the oil bath was increased to between 388–393 K and the reaction mixture refluxed at this temperature for 2 h. The reaction mixture was allowed to cool to room temperature and the reaction by-product (Me_3_SiCl) was removed *in vacuo*. The resulting crude white powder was recrystallized from a CH_2_Cl_2_/Hexane mixture producing colourless crystals. (Yield = 15.6 g, 91%).

### Refinement

Hydrogen atoms were placed in calculated positions with C—H distances ranging from 0.98 Å and included in the refinement in a riding-model approximation with *U*_iso_(H) = 1.5*U*_eq_(C). The positional parameters of the H atoms bonded to N atoms were refined independently and with *U*_iso_(H) = 1.5*U*_eq_(N). The N—H distances were constrained to be the same in each molecule [0.81 (2) Å] using the SADI command in *SHELXL* (Sheldrick, 2008).

### Crystal data

**Table:**

C_4_H_13_NO_2_SSi	*F*(000) = 360
*M**_r_* = 167.30	*D*_x_ = 1.275 Mg m^−^^3^
Monoclinic, *P*2_1_	Mo *K*α radiation, λ = 0.71073 Å
Hall symbol: P 2yb	Cell parameters from 6920 reflections
*a* = 8.2827 (4) Å	θ = 2.8–32.0°
*b* = 10.9513 (5) Å	µ = 0.45 mm^−^^1^
*c* = 9.6201 (3) Å	*T* = 150 K
β = 92.536 (2)°	Block, colourless
*V* = 871.75 (6) Å^3^	0.32 × 0.25 × 0.24 mm
*Z* = 4	

### Data collection

**Table:**

Nonius KappaCCD diffractometer	4894 independent reflections
Radiation source: fine-focus sealed tube	4195 reflections with *I* > 2σ(*I*)
graphite	*R*_int_ = 0.030
Detector resolution: 9 pixels mm^-1^	θ_max_ = 32.0°, θ_min_ = 2.8°
φ scans and ω scans with κ offsets	*h* = −12→12
Absorption correction: multi-scan (*SORTAV*; Blessing 1995)	*k* = −14→16
*T*_min_ = 0.830, *T*_max_ = 0.931	*l* = −12→14
6920 measured reflections	

### Refinement

**Table:**

Refinement on *F*^2^	Secondary atom site location: difference Fourier map
Least-squares matrix: full	Hydrogen site location: inferred from neighbouring sites
*R*[*F*^2^ > 2σ(*F*^2^)] = 0.039	H atoms treated by a mixture of independent and constrained refinement
*wR*(*F*^2^) = 0.094	*w* = 1/[σ^2^(*F*_o_^2^) + (0.0218*P*)^2^ + 0.660*P*] where *P* = (*F*_o_^2^ + 2*F*_c_^2^)/3
*S* = 1.05	(Δ/σ)_max_ < 0.001
4894 reflections	Δρ_max_ = 0.41 e Å^−^^3^
170 parameters	Δρ_min_ = −0.49 e Å^−^^3^
2 restraints	Absolute structure: Flack (1983), 1787 Friedel pairs
Primary atom site location: structure-invariant direct methods	Flack parameter: 0.39 (9)

### Special details

**Table:**

Geometry. All e.s.d.'s (except the e.s.d. in the dihedral angle between two l.s. planes) are estimated using the full covariance matrix. The cell e.s.d.'s are taken into account individually in the estimation of e.s.d.'s in distances, angles and torsion angles; correlations between e.s.d.'s in cell parameters are only used when they are defined by crystal symmetry. An approximate (isotropic) treatment of cell e.s.d.'s is used for estimating e.s.d.'s involving l.s. planes.
Refinement. Refinement of *F*^2^ against ALL reflections. The weighted *R*-factor *wR* and goodness of fit *S* are based on *F*^2^, conventional *R*-factors *R* are based on *F*, with *F* set to zero for negative *F*^2^. The threshold expression of *F*^2^ > σ(*F*^2^) is used only for calculating *R*-factors(gt) *etc*. and is not relevant to the choice of reflections for refinement. *R*-factors based on *F*^2^ are statistically about twice as large as those based on *F*, and *R*- factors based on ALL data will be even larger.

### Fractional atomic coordinates and isotropic or equivalent isotropic displacement parameters (Å^2^)

**Table:**

	*x*	*y*	*z*	*U*_iso_*/*U*_eq_	
S1A	0.84555 (7)	0.27915 (6)	0.95708 (6)	0.02134 (13)	
Si1A	0.55749 (8)	0.11459 (7)	0.99573 (7)	0.02169 (14)	
O1A	0.8724 (3)	0.40683 (18)	0.9815 (2)	0.0303 (4)	
O2A	0.8188 (2)	0.2381 (2)	0.81562 (18)	0.0308 (4)	
N1A	0.6945 (3)	0.2334 (2)	1.0411 (2)	0.0228 (4)	
H1NA	0.693 (4)	0.265 (3)	1.117 (2)	0.027*	
C1A	1.0156 (4)	0.2012 (3)	1.0271 (3)	0.0329 (6)	
H1AA	1.1115	0.2264	0.9783	0.049*	
H1AB	0.9994	0.1131	1.0158	0.049*	
H1AC	1.0307	0.2207	1.1262	0.049*	
C2A	0.6695 (4)	−0.0301 (3)	0.9698 (3)	0.0326 (6)	
H2AA	0.7328	−0.0505	1.0552	0.049*	
H2AB	0.7423	−0.0201	0.8930	0.049*	
H2AC	0.5926	−0.0960	0.9476	0.049*	
C3A	0.4308 (3)	0.1074 (3)	1.1496 (3)	0.0296 (6)	
H3AA	0.3737	0.1851	1.1595	0.044*	
H3AB	0.4997	0.0922	1.2332	0.044*	
H3AC	0.3520	0.0411	1.1374	0.044*	
C4A	0.4362 (4)	0.1526 (3)	0.8351 (3)	0.0322 (6)	
H4AA	0.3771	0.2288	0.8490	0.048*	
H4AB	0.3593	0.0866	0.8136	0.048*	
H4AC	0.5082	0.1626	0.7577	0.048*	
S1B	0.65985 (7)	0.31234 (6)	0.45178 (6)	0.02162 (13)	
Si1B	0.94662 (8)	0.47963 (7)	0.51424 (7)	0.02164 (14)	
O1B	0.6327 (3)	0.18445 (19)	0.4738 (2)	0.0310 (5)	
O2B	0.6801 (2)	0.3547 (2)	0.31117 (19)	0.0307 (4)	
N1B	0.8160 (3)	0.3552 (2)	0.5432 (2)	0.0223 (4)	
H1NB	0.820 (3)	0.317 (3)	0.615 (2)	0.027*	
C1B	0.4928 (4)	0.3904 (3)	0.5154 (3)	0.0340 (7)	
H1BA	0.3947	0.3668	0.4613	0.051*	
H1BB	0.5096	0.4787	0.5068	0.051*	
H1BC	0.4811	0.3694	0.6134	0.051*	
C2B	0.8316 (4)	0.6243 (3)	0.5137 (3)	0.0329 (6)	
H2BA	0.7775	0.6327	0.6018	0.049*	
H2BB	0.7506	0.6239	0.4363	0.049*	
H2BC	0.9057	0.6930	0.5027	0.049*	
C3B	1.0911 (3)	0.4706 (3)	0.6667 (3)	0.0309 (6)	
H3BA	1.0326	0.4805	0.7523	0.046*	
H3BB	1.1719	0.5355	0.6607	0.046*	
H3BC	1.1451	0.3910	0.6677	0.046*	
C4B	1.0488 (3)	0.4616 (3)	0.3477 (3)	0.0304 (6)	
H4BA	0.9686	0.4671	0.2699	0.046*	
H4BB	1.1023	0.3818	0.3460	0.046*	
H4BC	1.1294	0.5264	0.3392	0.046*	

### Atomic displacement parameters (Å^2^)

**Table:**

	*U*^11^	*U*^22^	*U*^33^	*U*^12^	*U*^13^	*U*^23^
S1A	0.0233 (3)	0.0193 (3)	0.0216 (3)	−0.0012 (2)	0.0028 (2)	0.0028 (2)
Si1A	0.0216 (3)	0.0221 (4)	0.0213 (3)	−0.0027 (3)	0.0008 (2)	−0.0005 (3)
O1A	0.0340 (11)	0.0163 (10)	0.0406 (11)	−0.0031 (8)	0.0019 (9)	0.0038 (9)
O2A	0.0417 (11)	0.0324 (11)	0.0187 (8)	−0.0058 (9)	0.0065 (7)	0.0011 (8)
N1A	0.0264 (10)	0.0239 (12)	0.0184 (10)	−0.0044 (9)	0.0032 (8)	−0.0033 (9)
C1A	0.0266 (13)	0.0273 (16)	0.0448 (17)	0.0039 (11)	0.0010 (12)	0.0066 (13)
C2A	0.0354 (15)	0.0216 (15)	0.0409 (15)	−0.0002 (11)	0.0039 (12)	−0.0032 (13)
C3A	0.0254 (12)	0.0378 (16)	0.0258 (12)	−0.0083 (11)	0.0038 (9)	−0.0004 (12)
C4A	0.0308 (14)	0.0392 (18)	0.0258 (13)	−0.0014 (11)	−0.0066 (10)	−0.0018 (12)
S1B	0.0240 (3)	0.0198 (3)	0.0211 (3)	−0.0010 (2)	0.0015 (2)	−0.0029 (2)
Si1B	0.0230 (3)	0.0216 (4)	0.0205 (3)	−0.0035 (3)	0.0030 (2)	0.0007 (3)
O1B	0.0349 (11)	0.0190 (11)	0.0387 (11)	−0.0052 (8)	−0.0021 (8)	−0.0036 (9)
O2B	0.0388 (11)	0.0333 (11)	0.0200 (8)	−0.0036 (8)	0.0005 (7)	−0.0021 (8)
N1B	0.0258 (10)	0.0209 (11)	0.0201 (10)	−0.0035 (8)	0.0007 (8)	0.0041 (9)
C1B	0.0244 (13)	0.0339 (18)	0.0443 (17)	0.0034 (11)	0.0060 (11)	−0.0082 (14)
C2B	0.0368 (15)	0.0209 (14)	0.0415 (16)	−0.0004 (12)	0.0057 (12)	0.0003 (13)
C3B	0.0277 (13)	0.0395 (17)	0.0253 (12)	−0.0071 (12)	−0.0015 (10)	0.0003 (13)
C4B	0.0321 (14)	0.0340 (17)	0.0260 (13)	−0.0037 (11)	0.0106 (10)	0.0026 (12)

### Geometric parameters (Å, °)

**Table:**

S1A—O1A	1.434 (2)	S1B—O1B	1.436 (2)
S1A—O2A	1.4410 (19)	S1B—O2B	1.4469 (19)
S1A—N1A	1.600 (2)	S1B—N1B	1.602 (2)
S1A—C1A	1.755 (3)	S1B—C1B	1.759 (3)
Si1A—N1A	1.768 (2)	Si1B—N1B	1.769 (2)
Si1A—C4A	1.853 (3)	Si1B—C2B	1.849 (3)
Si1A—C3A	1.853 (3)	Si1B—C3B	1.854 (3)
Si1A—C2A	1.859 (3)	Si1B—C4B	1.855 (3)
N1A—H1NA	0.81 (2)	N1B—H1NB	0.81 (2)
C1A—H1AA	0.9800	C1B—H1BA	0.9800
C1A—H1AB	0.9800	C1B—H1BB	0.9800
C1A—H1AC	0.9800	C1B—H1BC	0.9800
C2A—H2AA	0.9800	C2B—H2BA	0.9800
C2A—H2AB	0.9800	C2B—H2BB	0.9800
C2A—H2AC	0.9800	C2B—H2BC	0.9800
C3A—H3AA	0.9800	C3B—H3BA	0.9800
C3A—H3AB	0.9800	C3B—H3BB	0.9800
C3A—H3AC	0.9800	C3B—H3BC	0.9800
C4A—H4AA	0.9800	C4B—H4BA	0.9800
C4A—H4AB	0.9800	C4B—H4BB	0.9800
C4A—H4AC	0.9800	C4B—H4BC	0.9800
			
O1A—S1A—O2A	118.36 (12)	O1B—S1B—O2B	118.44 (12)
O1A—S1A—N1A	110.00 (13)	O1B—S1B—N1B	109.44 (12)
O2A—S1A—N1A	106.77 (12)	O2B—S1B—N1B	107.15 (12)
O1A—S1A—C1A	107.19 (15)	O1B—S1B—C1B	106.99 (15)
O2A—S1A—C1A	107.34 (15)	O2B—S1B—C1B	107.15 (15)
N1A—S1A—C1A	106.61 (14)	N1B—S1B—C1B	107.15 (14)
N1A—Si1A—C4A	111.04 (13)	N1B—Si1B—C2B	110.00 (13)
N1A—Si1A—C3A	102.36 (12)	N1B—Si1B—C3B	102.23 (12)
C4A—Si1A—C3A	111.74 (14)	C2B—Si1B—C3B	111.25 (15)
N1A—Si1A—C2A	109.97 (13)	N1B—Si1B—C4B	111.06 (13)
C4A—Si1A—C2A	109.58 (15)	C2B—Si1B—C4B	110.09 (15)
C3A—Si1A—C2A	111.98 (15)	C3B—Si1B—C4B	112.00 (13)
S1A—N1A—Si1A	127.83 (14)	S1B—N1B—Si1B	128.59 (14)
S1A—N1A—H1NA	112 (2)	S1B—N1B—H1NB	109 (2)
Si1A—N1A—H1NA	120 (2)	Si1B—N1B—H1NB	122 (2)
S1A—C1A—H1AA	109.5	S1B—C1B—H1BA	109.5
S1A—C1A—H1AB	109.5	S1B—C1B—H1BB	109.5
H1AA—C1A—H1AB	109.5	H1BA—C1B—H1BB	109.5
S1A—C1A—H1AC	109.5	S1B—C1B—H1BC	109.5
H1AA—C1A—H1AC	109.5	H1BA—C1B—H1BC	109.5
H1AB—C1A—H1AC	109.5	H1BB—C1B—H1BC	109.5
Si1A—C2A—H2AA	109.5	Si1B—C2B—H2BA	109.5
Si1A—C2A—H2AB	109.5	Si1B—C2B—H2BB	109.5
H2AA—C2A—H2AB	109.5	H2BA—C2B—H2BB	109.5
Si1A—C2A—H2AC	109.5	Si1B—C2B—H2BC	109.5
H2AA—C2A—H2AC	109.5	H2BA—C2B—H2BC	109.5
H2AB—C2A—H2AC	109.5	H2BB—C2B—H2BC	109.5
Si1A—C3A—H3AA	109.5	Si1B—C3B—H3BA	109.5
Si1A—C3A—H3AB	109.5	Si1B—C3B—H3BB	109.5
H3AA—C3A—H3AB	109.5	H3BA—C3B—H3BB	109.5
Si1A—C3A—H3AC	109.5	Si1B—C3B—H3BC	109.5
H3AA—C3A—H3AC	109.5	H3BA—C3B—H3BC	109.5
H3AB—C3A—H3AC	109.5	H3BB—C3B—H3BC	109.5
Si1A—C4A—H4AA	109.5	Si1B—C4B—H4BA	109.5
Si1A—C4A—H4AB	109.5	Si1B—C4B—H4BB	109.5
H4AA—C4A—H4AB	109.5	H4BA—C4B—H4BB	109.5
Si1A—C4A—H4AC	109.5	Si1B—C4B—H4BC	109.5
H4AA—C4A—H4AC	109.5	H4BA—C4B—H4BC	109.5
H4AB—C4A—H4AC	109.5	H4BB—C4B—H4BC	109.5

### Hydrogen-bond geometry (Å, °)

**Table:**

*D*—H···*A*	*D*—H	H···*A*	*D*···*A*	*D*—H···*A*
N1B—H1NB···O2A	0.81 (2)	2.11 (2)	2.917 (3)	173 (3)
N1A—H1NA···O2B^i^	0.81 (2)	2.12 (2)	2.925 (3)	177 (3)

Symmetry codes: (i) *x*, *y*, *z*+1.
